# Supplementary material for: The self-healing of defects induced by the hydriding phase transformation in palladium nanoparticles
Source: Nat Commun. 2017 Nov 9;8:1376. doi: 10.1038/s41467-017-01548-7 (PMC5680230; doi:10.1038/s41467-017-01548-7)
Supplement: Supplementary file 1 — Supporting Information [file 41467_2017_1548_MOESM1_ESM.pdf]

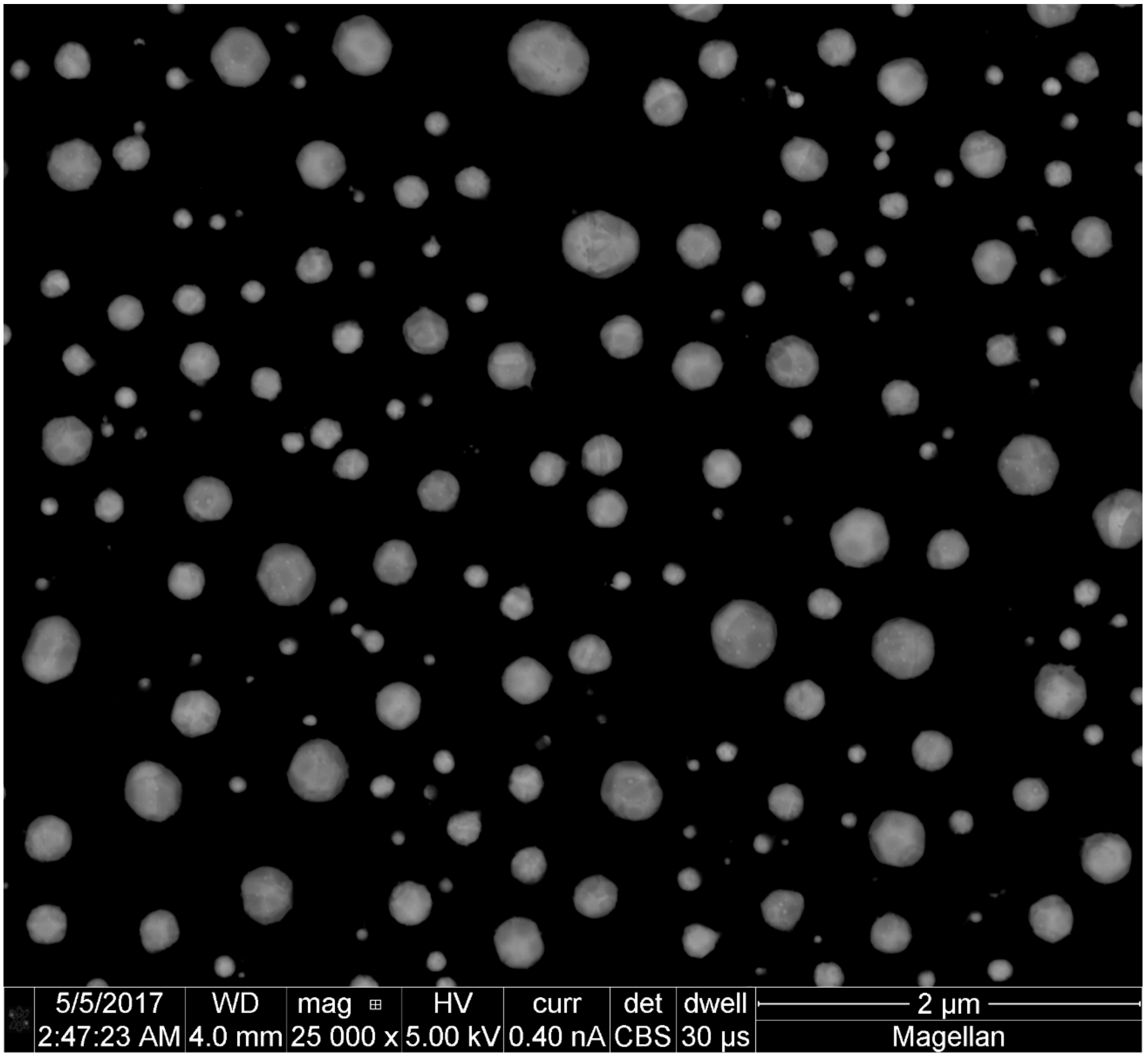

Supplementary Figure 1. **Electron microscopy image of the as-synthesized Pd nanoparticles**. The particles tend to be roughly spherical in shape.

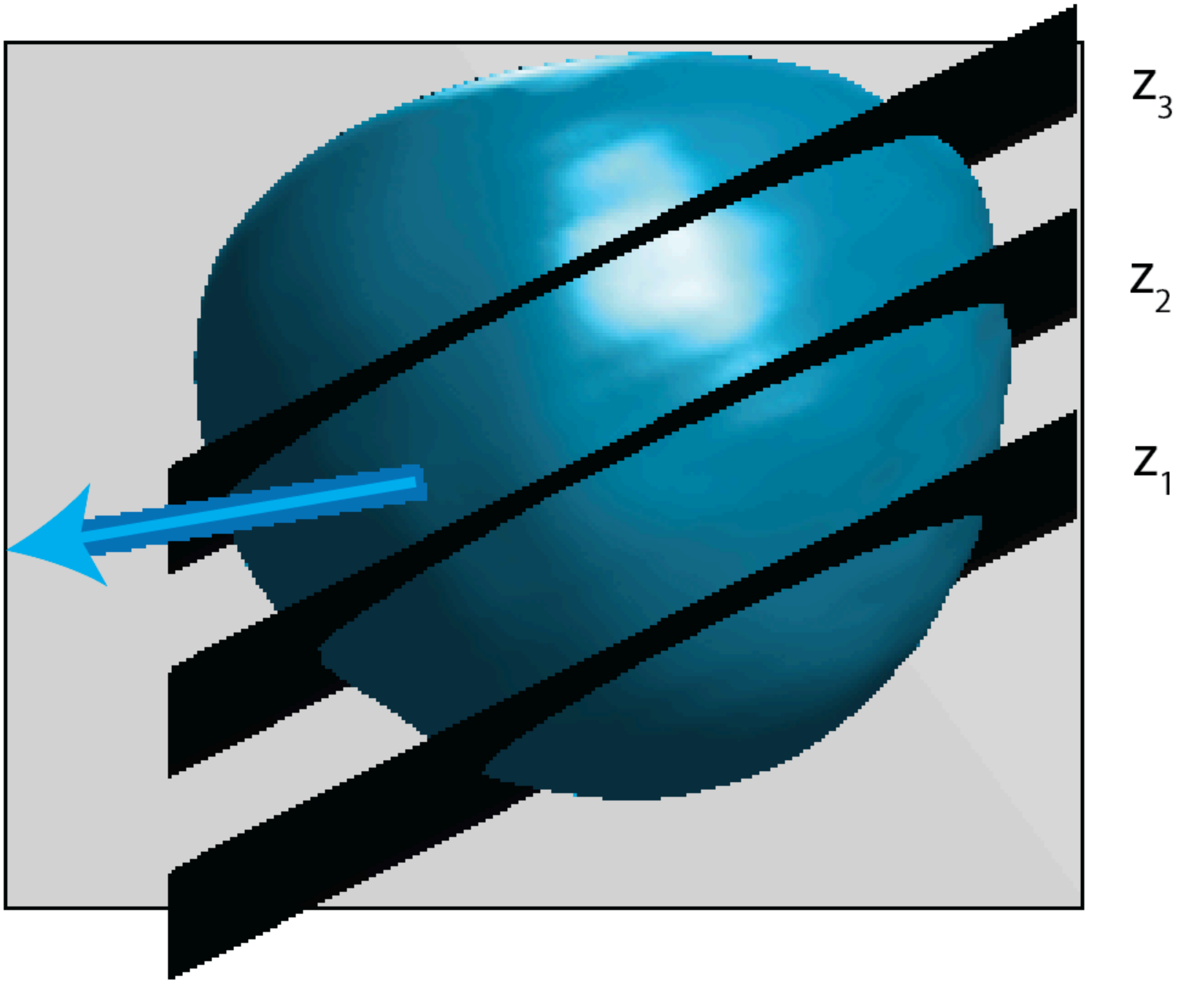

Supplementary Figure 2. **Cross-section locations for the cross-sections shown in Figure 1**. The measured scattering vector is shown as a blue arrow.

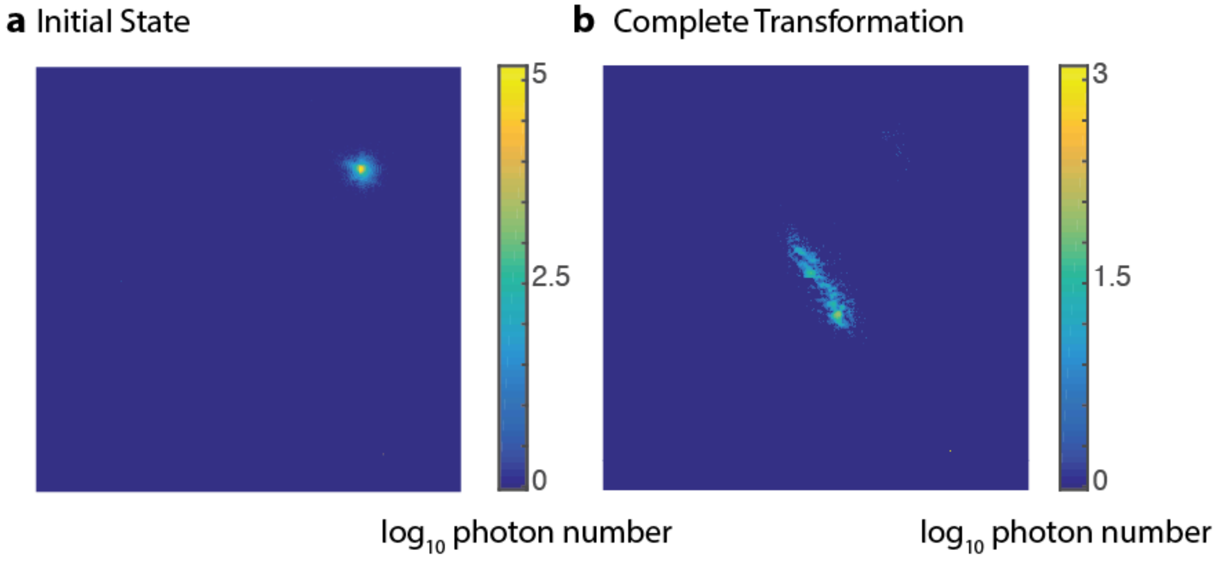

Supplementary Figure 3. Coherent diffraction data for Pd particle discussed in the main text. (**a**) The as-synthesized state. **(b**) After the complete transformation. In **b** there is a lack of centrosymmetry, a clearly defined central maximum, and the spread in the diffraction pattern is much larger than in **a**. All of these features are consistent with a highly defective particle.

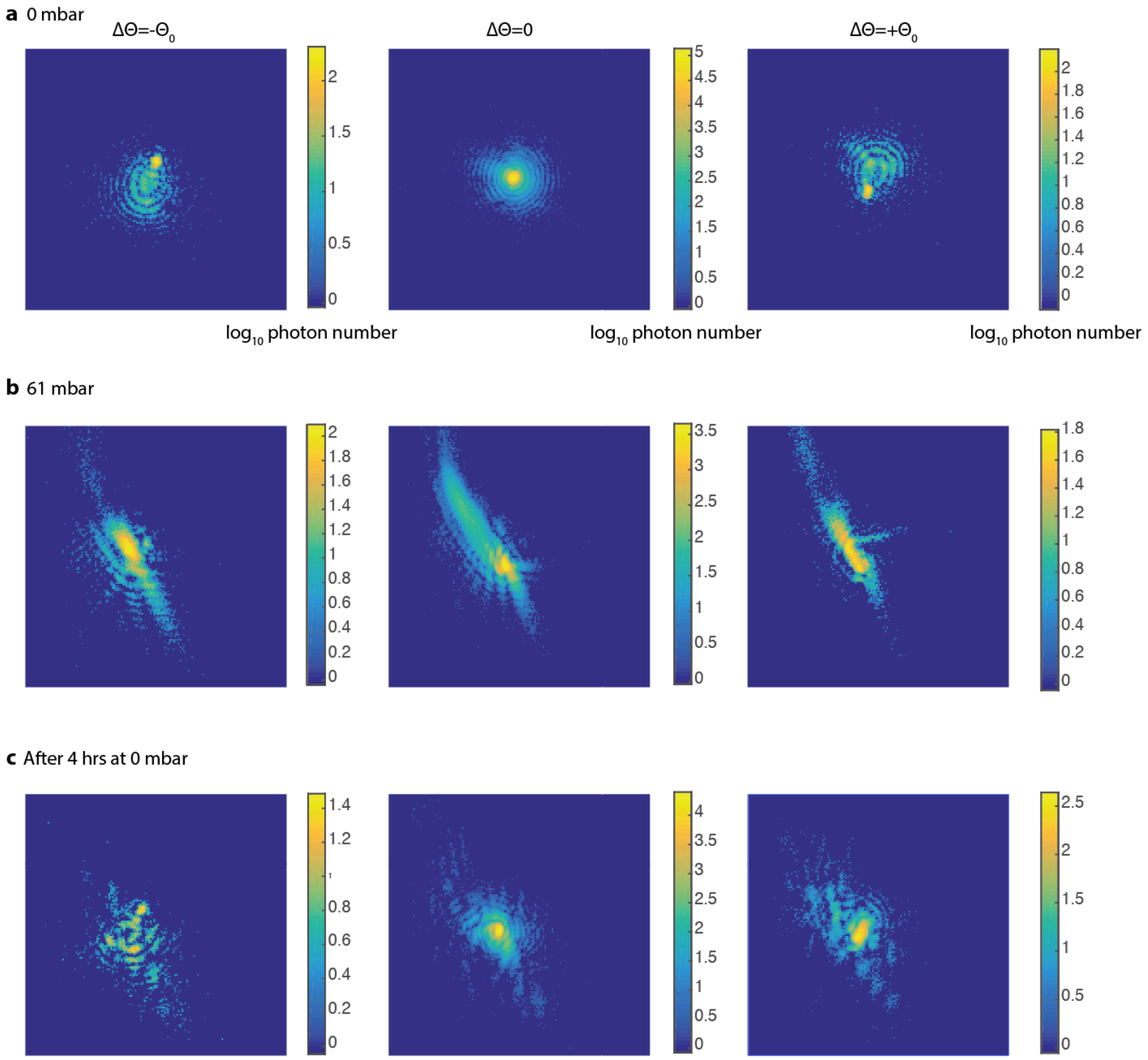

Supplementary Figure 4. Coherent diffraction data used for the reconstructions shown in Figure 1. Three cross-sections are shown. The nucleation and healing of the defects are evident in the diffraction data. (**a**) The Pd nanoparticle diffraction data in the as-synthesized state. **(b**) The Pd nanoparticle diffraction data at 61 mbar pH_2. (**c**) The Pd nanoparticle diffraction data at 0 mbar pH_2 after 4 hours.

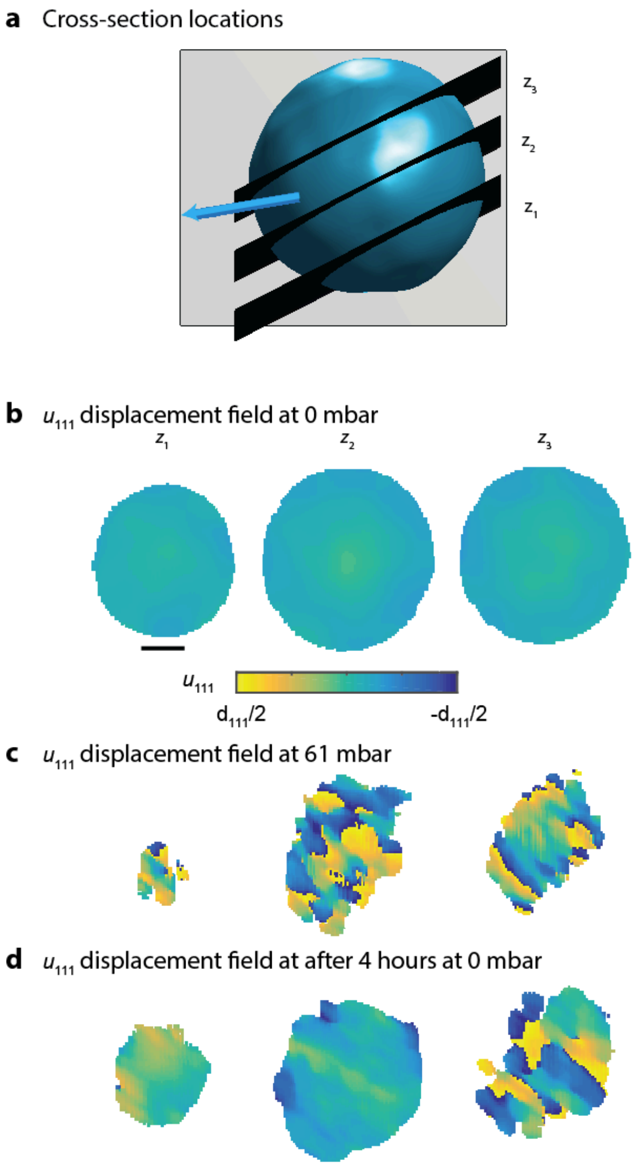

Supplementary Figure 5. Another Pd nanoparticle that shows defect nucleation and subsequent healing. The scale bar represents 100 nm. (**a**) The spatial locations of the cross-sections shown in **b-d**. **(b**) Three cross-sections showing the as-synthesized displacement field. The particle is nearly strain-free and is free of dislocations. (**c)** Cross-sections showing defects induced in the particle center due to the partial transformation at 61 mbar. (**d)** Cross-sections showing defects healing in the particle center due to 4 hours at 0 mbar. The defects have healed in cross-section z_2 and some remain in cross-section z_3.

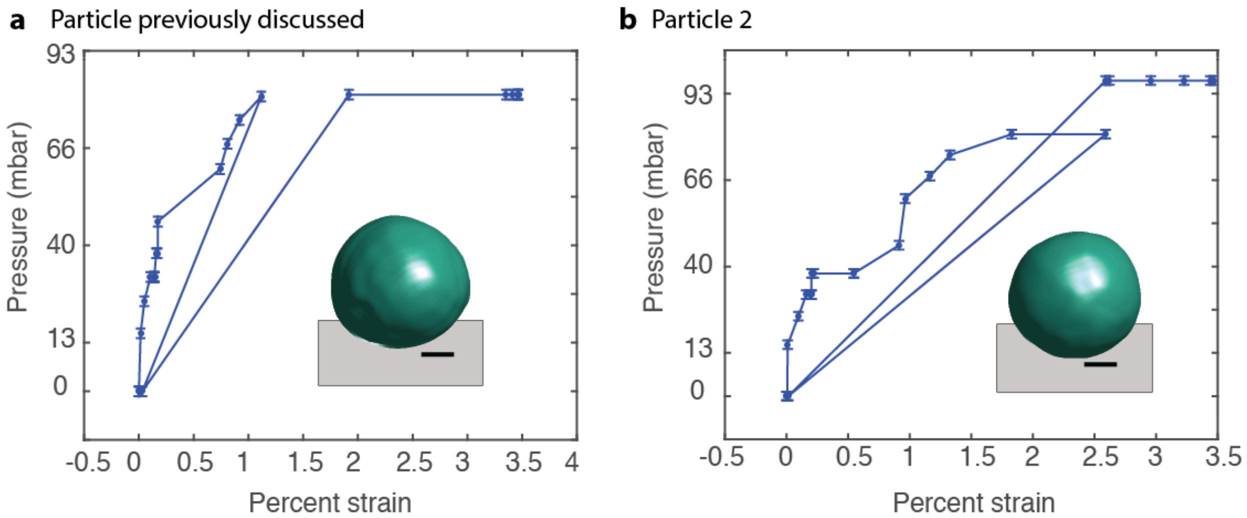

Supplementary Figure 6. Pressure-strain isotherms for the particles discussed in the main text. The scale bar represents 100 nm. The return to 0 mbar is shown in this Figure. Error bars show the average drift in the pressure gauge over the course of the measurement.

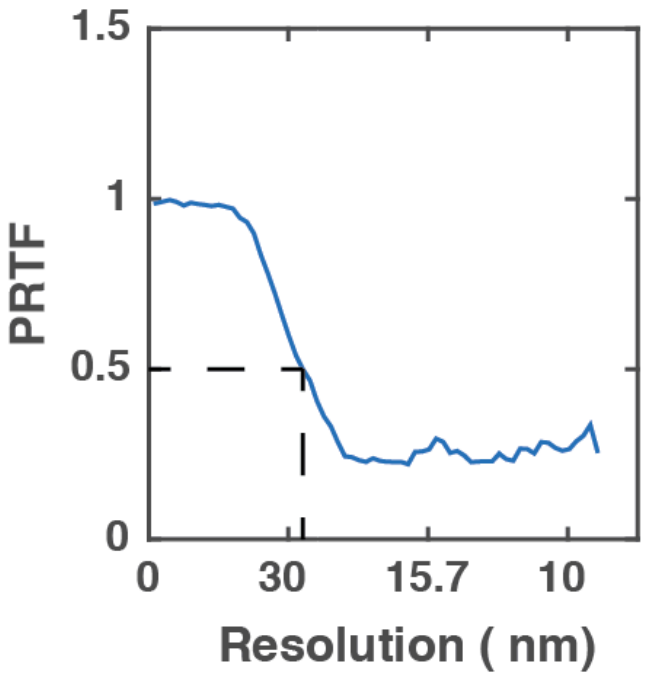

Supplementary Figure 7. The phase retrieval transfer function. A conservative cutoff of 0.5 is used to estimate the resolution as between 20-30 nm.
