# Supplementary material for: The self-healing of defects induced by the hydriding phase transformation in palladium nanoparticles
Source: Nat Commun. 2017 Nov 9;8:1376. doi: 10.1038/s41467-017-01548-7 (PMC5680230; doi:10.1038/s41467-017-01548-7)
Supplement: Supplementary file 3 — Description of Additional Supplementary Information [file 41467_2017_1548_MOESM3_ESM.pdf]

Description of Additional Supplementary Files

File Name: Supplementary Movie 1
Description: Dislocation Network at 61 mbar H_2 pressure. Caption: The particle shape is shown by the black isosurface while the dislocation lines are shown in red. The state shown was measured at 61 mbar of H_2 pressure. The dislocation network extends through the particle from the surface into the bulk.

File Name: Supplementary Data 1
Description: Dislocation Network at 81 mbar H_2 pressure. Caption: The particle shape is shown by the black isosurface while the dislocation lines are shown in red. The state shown was measured at 81 mbar of H_2 pressure. The dislocation network extends through the particle from the surface into the bulk.

File Name: Supplementary Software 1
Description: Dislocation Network after 4 hours at 0 mbar H_2 pressure. Caption: The particle shape is shown by the black isosurface while the dislocation lines are shown in red. The state shown was measured after 4 hours at 0 mbar of H_2 pressure. The dislocation network has healed and only a few dislocations near the surface remain.
